# Implementation and survey evaluation of a new safety concern escalation pathway among nursing team members

**DOI:** 10.1371/journal.pone.0333736

**Published:** 2025-10-27

**Authors:** Indica Sur, Rocel D. Besa, Stephanie Ceylan, Brett Sealove, Elliot Frank

**Affiliations:** 1 Hackensack Meridian School of Medicine, Nutley, New Jersey, United States of America; 2 Department of Nursing Quality & Professional Practice, Jersey Shore University Medical Center, Hackensack Meridian *Health*, Neptune, New Jersey, United States of America; 3 Department of Emergency Department Management Services, Nursing Administration, Jersey Shore University Medical Center, Hackensack Meridian *Health*, Neptune, New Jersey, United States of America; 4 Department of Cardiology, Jersey Shore University Medical Center, Hackensack Meridian *Health*, Neptune, New Jersey, United States of America; 5 Department of Patient Safety & Quality, Jersey Shore University Medical Center, Hackensack Meridian *Health*, Neptune, New Jersey, United States of America; Alexandria University Faculty of Nursing, EGYPT

## Abstract

**Introduction:**

Escalating patient safety concerns is a critical component of healthcare delivery and can be impeded by organizational barriers including power hierarchies and fear of retaliation. Nursing team members are pivotal in identifying and addressing real-time safety issues. The existing literature on increasing “speaking-up” behaviors among nursing team members predominantly focuses on communication training rather than simplifying the escalation process. The current project aimed to evaluate the awareness and perceptions of a newly implemented pathway to streamline concern escalation among nursing team members at a tertiary academic medical center. Although the pathway was embraced and regularly utilized by nursing supervisors, we sought to determine whether front-line staff were aware of, and confident in, the use of the pathway.

**Methods:**

A new escalation pathway for communicating concerns was developed and implemented in response to a patient safety event and subsequent root cause analysis. An investigator-developed electronic survey was distributed to nursing team members and assessed (1) demographic data, (2) experiences with escalating safety concerns, and (3) awareness, use, and attitudes toward the new pathway. Recruitment and data collection occurred in two stages, and responses were collected via REDCap. Data were analyzed using descriptive statistics and qualitative thematic analysis of free-text response items.

**Results:**

128 of 129 total responses were analyzed. Of 89 (69.5%) participants who indicated they had ever encountered a situation requiring escalation, 48 of them (53.9%; 37.5% of all respondents) reported hesitating to act because they were uncertain how to escalate the concern. 25.6% of respondents had already used the new pathway for escalating concerns, and 79% thought they would use the pathway in the future. However, 41% expressed ambivalence about its impact on their confidence in escalating concerns and 47% were still unsure how to use the matrix/pathway. Thematic analysis of 51 free-text response items identified five key themes which provided useful provided useful insights to guide ongoing education and support for the escalation process.

**Conclusion:**

The new escalation pathway was positively received and improved confidence for many, though some gaps in awareness and training persist.

## Introduction

Approximately 1 in 10 patients face a preventable safety concern while hospitalized [1], and communication error between healthcare providers is cited as a primary contributor to these adverse events [2–5]. Identifying and escalating concerns about patient safety–sometimes referred to as “speaking-up” behaviors [2]–has been identified as a critical tool in mitigating adverse events and improving safety outcomes [3,6–10]. Nursing and other frontline team members are often most closely attuned to patients’ real-time status and play an essential role in promoting and protecting patient safety [5,11]. Unfortunately, team members often encounter critical situations where they may (1) struggle to reach the provider responsible for a patient in decline, or (2) worry that a provider’s orders are inappropriate or pose a risk to patient safety. It is essential that these team members can rapidly and effectively escalate patient safety concerns.

Many factors impact willingness to speak up, however, including organizational culture, power hierarchies, perceived lack of impact, and fear of retribution [4,6,7,9,12,13]. Additionally, nursing team members specifically report feeling less empowered to voice concerns than other healthcare providers, have less favorable views of patient safety culture, and face significant barriers to engaging in quality improvement initiatives [4,6,9–12,14]. Designing effective, targeted interventions to promote speaking-up behaviors among front-line team members is essential to delivering high-quality care and improving patient outcomes [10,11,15].

Much of the prior work in this area targets interprofessional and interpersonal communication strategies and conflict resolution techniques. Examples of these interventions include scenario-based training for nurses to practice speaking up in response to potentially dangerous provider treatments [16] and a communication-based algorithm designed to help promote assertiveness in real-time conflict escalation in interprofessional teams [17]. Despite significant work implementing communication-based interventions, the efficacy of these strategies has been suboptimal. Several meta-analyses, including two systematic reviews [15,18], two scoping reviews [19,20], and a systematic narrative review [8] failed to show consistent efficacy of these interventions. Even seemingly effective techniques may be thwarted by organizational culture and emergent situations [8].

Finally, while several projects have evaluated perceived barriers to speaking-up behaviors in nursing staff [4,11,20], only a few studies have addressed escalation beyond the immediate care team or collected direct feedback from team members [4,9,13].

Given the critical importance of speaking-up behaviors, the significant potential barriers to concern escalation, and the relative inconsistency of communication-skills based intervention strategies, alternative intervention strategies and further insight into nursing team members’ perspectives on these initiatives are clearly needed. The current project aimed to fill these gaps by presenting a simple alternative strategy which facilitates escalation beyond the direct patient care team coupled with an analysis of nurses’ attitudes and awareness of the new model.

### Preceding event

**Fig 1:**
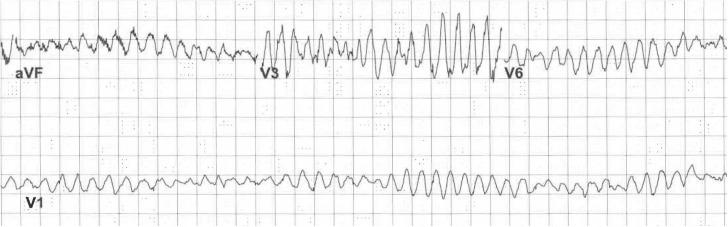
Adverse Event. Patient’s electrocardiogram showing dysrhythmia after receiving contraindicated medication.

**Fig 2:**
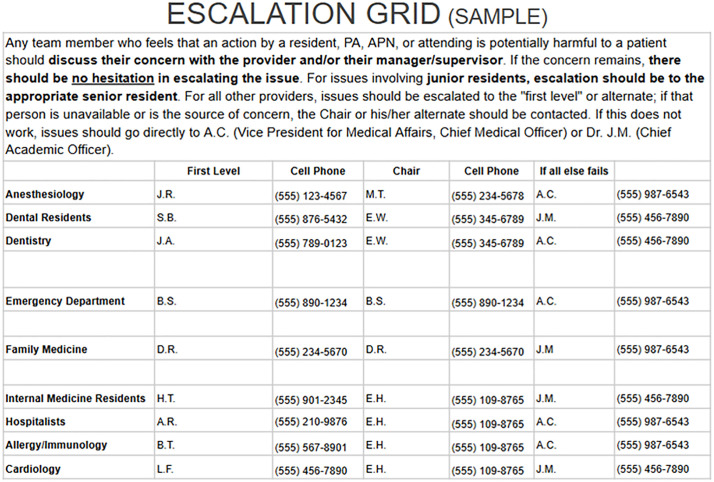
Escalation Matrix. Sample matrix (names and phone numbers are fictitious) with explanation for use. “A.C.” is the Vice President for Medical Affairs/ Chief Medical Officer and “J.M.” is the Chief Academic Officer for the hospital.

A quality review was conducted after a patient received dofetilide despite a prolonged QTc, resulting in a life-threatening dysrhythmia (Fig 1). The review revealed that both the nursing and pharmacy staff were aware of the contraindication and emphatically communicated their concern to the prescribing cardiologist, who insisted on administering the medication. The review team concluded that the root cause of the event was the absence of a precise mechanism for the nurses to escalate their concerns in real-time. In response to this event, an interprofessional team developed a simplified concern escalation algorithm, delineating a series of individuals, with their contact information, who can be reached in real-time by bedside team members with patient safety concerns (Fig 2). The plan was embraced by all Department Chairs and Section Chiefs and endorsed by the hospital’s Medical Executive Committee. The completed pathway was distributed to all nursing supervisors and each unit within a single large hospital, and all nursing units received training on the pathway. Copies of the pathway (without phone numbers) were displayed at all Nursing Stations; all Nursing Supervisors carried full copies including phone numbers.

### Study aims

Although the guideline was communicated through regular nursing channels and all nursing supervisors expressed their knowledge of and appreciation for the new paradigm, it was still unclear if frontline team members were aware of the pathway and if this had impacted their confidence in escalating issues. We electronically surveyed nursing team members to determine (1) whether they were aware of the new escalation matrix, and (2) if they felt confident in using the matrix.

## Methods

### Study setting

This study was conducted within a 691-bed non-profit, tertiary research and academic medical center located in the Northeastern United States.

### Study population and sampling

The target population consisted of all Nursing Team members at the hospital, including registered nurses (RNs), licensed practical nurses (LPNs), nurse managers, assistant nurse managers, patient-care technicians (PCTs), and certified nursing assistants (CNAs). To be eligible, participants were required to have an active affiliation (employed or through agency) with the hospital on any emergency or inpatient unit; at the time of this project, there were approximately 1400 eligible team members. The survey was distributed as an electronic link and QR code via email and standardized group messages, flyers posted in nursing department break rooms, and in-person during team huddles and council/committee meetings.

### Survey design

The investigator-developed survey contained 16 questions categorized into three main sections:

(1) Demographic information including team member role, work status (full-time, part-time, per-diem, agency/traveler), and duration of affiliation.
(2) Questions specific to team members’ experiences with needing to escalate concerns and their awareness and utilization of the matrix.
(3) Team members’ attitudes towards the matrix which included an opportunity for free text comments.

The survey was piloted for the first week of data collection involving an initial set of 21 responses. No iterative changes were deemed necessary; therefore, the piloted data was retained in the final analysis.

### Data collection

Initial data collection began approximately twelve months after implementation of the escalation pathway and the survey remained open for one month. Due to a low response rate, the decision was made to conduct a second phase of recruitment seven months later, allowing the survey to remain open for a total of six months. All study data were collected and managed using REDCap (Research Electronic Data Capture) [21,22]. Data collection began on 02/17/2023 and concluded on 06/13/2024.

### Data analysis

Survey results were tabulated, and descriptive statistics were used to describe respondents’ work status, role, and length of employment. Forced-choice questions about the participant’s experiences with concern escalation as well as awareness of and attitudes towards the matrix, were similarly analyzed. Inductive thematic analysis of free-response questions was conducted using the methodology for qualitative data described by Braun and Clarke [23]. This methodology involves six phases of analysis: gaining familiarity with the data, generating initial codes, searching for themes, reviewing themes, defining and naming themes, and producing the report. All free-response data were coded by one study team member trained on the methodology for consistency. Descriptive statistics of all data were calculated using Excel software.

### Study rigor

The survey was created following a literature review for relevance using multiple question types (multiple-choice, Likert-scale, free response). Given the research aim of evaluating awareness and attitudes towards the matrix, it was determined that this mixed-methods approach would be most appropriate to capture nursing team members’ perspectives. The survey design was reviewed independently by study team members and was piloted for data collection without iterative refinement. Participant recruitment occurred throughout all units to capture diverse nursing roles and backgrounds. Thematic analysis was conducted following the widely accepted methodology described by Braun and Clarke [23].

### Ethics considerations

The Hackensack Meridian *Health* Institutional Review Board (HIRB) provided ethical oversight and approved all survey and recruitment materials before the study started (Pro2022−0663). Due to the nature of the online survey project, the HIRB also approved a waiver of consent documentation prior to data collection. The consent form was displayed as the first item of the survey; to proceed with the survey, participants indicated they read the information provided and agreed to the following statement: “By completing this survey, I indicate that I have read the information provided and agree to participate.” To maintain confidentiality during data collection and analysis, no personal identifiers were collected with survey responses. Any potentially identifying information submitted in the free response (e.g., unit name) was removed prior to thematic analysis.

## Results

Out of all those asked to participate in the survey, approximately 9% responded. Of 129 responses, 128 responses met the criteria and were analyzed. Demographic data are displayed below (Table 1).

**Table 1:** Demographic data.

Role	Frequency	Percent (%)
RN	98	76.6
PCT or CNA	3	2.3
Assistant Nurse Manager	19	14.8
Nurse Manager	8	6.3
**Work-status**	**Frequency**	**Percent (%)**
Full-time	110	85.94
Part-time	5	3.91
Per-diem	11	8.59
Agency/Traveler	2	1.56
**Employment duration**	**Frequency**	**Percent (%)**
0-1 year	34	26.56
2-4 years	27	21.09
5-7 years	14	10.94
8 + years	53	41.41

Breakdown of respondents’ demographic data including current role, work-status, and employment duration at the medical center.

89 respondents (69.5%) had been in a situation where they were concerned a provider’s actions could harm a patient. 48 (53.93%) of these (37.5% of total respondents) indicated that they had also hesitated to act on these concerns because they did not know who to contact beyond the provider and their immediate supervisor. Importantly, this trend was seen across all lengths of employment.

34 respondents (25.56%) indicated they had already used the escalation pathway/matrix and 113 (88.28%) indicated they would feel comfortable using the matrix in the event of a patient safety concern. Overall, respondents were aware of, and positive about, the pathway but these feelings were not universal (Table 2).

**Table 2:** Likert-scale responses.

Prompt Statement	Response Frequency and Percent (%)				
	Strongly Agree	Slightly Agree	Neither Agree nor Disagree	Slightly Disagree	Slightly Agree
The Escalation Path has improved my confidence in reporting concerns regarding patient safety.	29 (22.66%)	30 (23.44%)	**53 (41.41%)**	7 (5.47%)	9 (7.03%)
I am not sure how to use the Escalation Path.	**32 (25.40%)**	27 (21.43%)	20 (15.87%)	18 (14.29%)	29 (23.01%)
I feel the Escalation Path has clarified my understanding of how to escalate concerns regarding patient safety.	**43 (34.13%)**	29 (23.01%)	39 (30.95%)	6 (4.76%)	9 (7.14%)
If a situation arose where I was concerned that a providers’ actions could potentially harm a patient, I would refer to the Escalation Path to escalate the issue.	**68 (53.54%)**	32 (25.19%)	19 (14.96%)	2 (1.56%)	6 (4.72%)
If a situation arose where I was concerned that a providers’ actions could potentially harm a patient, there is another individual I would prefer to contact, after the provider in question or my immediate supervisor, rather than one outlined in the Escalation Path.	23 (17.97%)	26 (20.31%)	**59 (46.09%)**	12 (9.36%)	8 (6.25%)

Five-point ordinal Likert-scale statements and frequency of nursing team members’ responses.

Almost half of the respondents included comments following the free-text prompt and five overall themes were identified: (1) Positive, (2) Unsure, (3) Negative, (4) Specific Concern, and (5) Other, examples of which are tabulated below (Table 3).

**Table 3:** Themes within free-text responses.

Theme	Frequency (Percent)	Sample Responses
Positive	21 (41.18%)	*I think it is helpful to the department for after hours procedures* *I think the process brings greater clarity to elevating issues.* *I think this has been a great asset in our toolkits.*
Unsure	17 (33.36%)	*I have not learned or used this yet.* *I was not educated on the escalation path.*
Negative	7 (13.73%)	*In my opinion the Escalation Path does not work in [this] department.* *Would address [concerns] to manager if necessary.*
Specific Concern	2 (3.92%)	*Concerns are with how the situation is handled once the information is received.* *My concern is always retaliation from the provider after voicing concern. My most recent incident did not result in retaliatory action at all from the provider, which makes me trust this provider more.*
Other	5 (9.80%)	*How is this being educated to all staff?* *It would be helpful if this pathway could be emailed out to all team members for easier access.*

Theme frequency and direct quotations of free-text response items grouped by thematic analysis.

21 responses were “Positive,” and indicated they felt the matrix is a valuable tool for concern escalation and that they would be likely to use it in the future. 17 were “Unsure” and indicated they did not know about the matrix or desired additional training on the pathway. Seven were “Negative” and indicated they were not likely to use the matrix, doubted its efficacy, or would instead prefer to use another means of escalating a patient care concern. Two responses indicated a specific concern related to the potential for retaliation; both responses are included in Table 2 below. Finally, 5 responses did not fit another theme and included requests for wider distribution of the pathway.

## Discussion

This paper describes the implementation of a novel matrix designed to facilitate escalation of patient care concerns beyond the immediate care team and the assessment of nursing team members’ awareness of and attitudes towards it. The survey addressed the well-known need for improved concern escalation procedure with almost 70% of respondents having encountered situations where they believed a provider’s actions could potentially harm a patient and over half of those respondents encountering difficulty in escalation. Surprisingly, this uncertainty was reported across all lengths of employment, including team members with over eight years’ experience.

The overall response to the new escalation matrix in our hospital was overwhelmingly positive but still showed significant gaps in awareness of the pathway and confidence in its efficacy. Although there had been twelve months of implementation and multiple training sessions on the pathway prior to data collection, over one-third of participants indicated they had never heard of the pathway and nearly half were unsure about how to use it. These results suggest that the currently employed training methods may not have been consistent across all units or may not be effective for this type of intervention. Consistency of training and alternative training models will need to be explored.

Two responses specifically requested greater accessibility of the full matrix, through either email or print versions in each department. In the current system, the full matrix is held on each unit by the nursing managers and a version without phone numbers is posted in each department; our findings suggest that this paradigm may represent another unintentional barrier to its use or circulation. These responses also further highlight the importance of direct qualitative feedback from team members when evaluating such interventions, so such issues can be identified and addressed.

While 80% of respondents would refer to the matrix to escalate a future concern, nearly half of participants expressed ambivalence or uncertainty when asked if the pathway actually improved their confidence in escalating safety concerns. This finding reveals a concerning disconnect between attitudes towards the tool and team members’ confidence in its real-world efficacy. This was further highlighted by the “Specific Concern” theme identified within the free responses, with two respondents citing explicit fear of retaliation after using the matrix. The escalation pathway was widely supported by hospital administration and medical staff leadership, but this support may not have been clearly communicated in training sessions. While this concern was voiced buy a small fraction of respondents, it is potentially a significant cultural barrier to effective implementation of this tool and there is still work to be done to reassure front-line team members

In a recent Magnet survey of the institution, one reviewer commented they had never seen a tool like this and recommended it be expanded across other hospitals within and outside our health system. This feedback, along with the overwhelmingly positive attitudes towards the matrix, suggests that this simplified escalation procedure is a valuable tool though implementation and accessibility will require refinement.

### Implications

Our findings have several key implications for nursing teams and patient safety initiatives. First, communication skills-based training alone is insufficient to improve speaking-up behaviors; alternative interventions must be explored. This new matrix appears to be a simple and effective way to foster escalation in real time. However, improvement teams cannot assume that training and support for new processes will in fact be absorbed by all those targeted; training requires repetition and efficacy of training requires assessment. Finally, even in hospitals and health systems that have taken a strong stance against retaliation and promoted “speaking up for safety” as part of their culture, some front line staff may still be reticent to come forward; health systems must ensure that this culture truly permeates all levels of the organization.

### Strengths & Limitations

The current project has several unique features. While most previous work to increase speaking-up behaviors has focused on training communication skills [8,15,18–20], the current project sought to simplify and facilitate the process of concern escalation. Furthermore, by obtaining qualitative data about awareness of, and attitudes about, the new process, we were able to assess its efficacy and refine both the process and its implementation.

Our study has several limitations. First, survey data and voluntary response sampling are inherently subject to response bias, and those who participate may already be engaged with quality improvement initiatives or have dealt with escalation of patient safety concerns in the past. Despite numerous attempts to recruit participants, the response rate was smaller than we had anticipated, which may reflect a feeling of clerical overload in front-line staff. This limits the confidence in the conclusions which can be drawn regarding reception of the matrix among nursing team members. Finally, our work did not capture evidence of the actual use of the matrix, which would be highly beneficial in understanding its applicability and efficacy in real-world settings.

### Future directions

Expanding on this work, the researchers have since developed and launched an updated safety concern escalation matrix, the evaluation of which will be a future project. Other future work will focus on targeted education and training, evaluation of the use of the pathways in real-time, and affecting culture shifts to alleviate fears of retaliation. To foster successful implementation of this and other new processes, education could incorporate online, case-based training modules [24], multimodal training sessions within team huddles [25,26], or designated patient safety champions [27,28] to help facilitate trust and adoption of these initiatives.

Accessibility of the matrix was another potential barrier identified by our qualitative analysis. Future interventions could expand reach by integrating the pathway into the electronic health record or other employee-facing applications.

Finally, all organizations need to continue efforts to reassure staff about retaliation and adopt strategies that support a psychologically safe environment for concern escalation and increase speaking-up behaviors [9,10,15,19,29,30].

## Conclusions

Nursing team members frequently encounter patient safety concerns but may not know how to escalate concerns. A simple matrix can help foster escalation and appears to be well-received by front line staff but active education, training, and organizational support is required and lingering fears of retaliation need to be addressed.

## Supporting information

S1 File Final coded dataset (deidentified). (CSV)
